# Molecular Analysis of VanA Outbreak of *Enterococcus faecium* in Two Warsaw Hospitals: The Importance of Mobile Genetic Elements

**DOI:** 10.1155/2014/575367

**Published:** 2014-06-09

**Authors:** Ewa Wardal, Katarzyna Markowska, Dorota Żabicka, Marta Wróblewska, Małgorzata Giemza, Ewa Mik, Hanna Połowniak-Pracka, Agnieszka Woźniak, Waleria Hryniewicz, Ewa Sadowy

**Affiliations:** ^1^National Medicines Institute, ul. Chełmska 30/34, 00-725 Warsaw, Poland; ^2^Institute of Hematology and Transfusion Medicine, ul. Ghandi 14, 02-776 Warsaw, Poland; ^3^Institute of Oncology, ul. Roentgena 5, 02-781 Warsaw, Poland

## Abstract

Vancomycin-resistant *Enterococcus faecium* represents a growing threat in hospital-acquired infections. Two outbreaks of this pathogen from neighboring Warsaw hospitals have been analyzed in this study. Pulsed-field gel electrophoresis (PFGE) of *Sma*I-digested DNA, multilocus VNTR analysis (MLVA), and multilocus sequence typing (MLST) revealed a clonal variability of isolates which belonged to three main lineages (17, 18, and 78) of nosocomial *E. faecium*. All isolates were multidrug resistant and carried several resistance, virulence, and plasmid-specific genes. Almost all isolates shared the same variant of Tn*1546* transposon, characterized by the presence of insertion sequence IS*Ef1* and a point mutation in the *vanA* gene. In the majority of cases, this transposon was located on 50 kb or 100 kb pRUM-related plasmids, which lacked, however, the *axe-txe* toxin-antitoxin genes. 100 kb plasmid was easily transferred by conjugation and was found in various clonal backgrounds in both institutions, while 50 kb plasmid was not transferable and occurred solely in MT159/ST78 strains that disseminated clonally in one institution. Although molecular data indicated the spread of VRE between two institutions or a potential common source of this alert pathogen, epidemiological investigations did not reveal the possible route by which outbreak strains disseminated.

## 1. Introduction

Since the first isolation of vancomycin-resistant enterococci (VRE) in 1986 [1, 2], this phenotype has spread rapidly and now is present in hospitals worldwide [3]. In Poland, the first VanA outbreak took place in the adult hematology ward of Gdansk Medical University in December 1996, followed by outbreaks in other centers [4]. The predominant species among VRE is* Enterococcus faecium* (VR*Efm*). The majority of worldwide VR*Efm* belongs to the meroclone CC17 (ciprofloxacin- and ampicillin-resistant and enriched in putative virulence traits), recently split into three distinct lineages, 17, 18, and 78, that evolved in hospital environment through horizontal gene transfer (HGT) and recombination processes [5]. These hospital-adapted lineages play a crucial role in the emergence and spread of VR*Efm*.

The* vanA* gene cluster is a widely studied vancomycin/teicoplanin resistance determinant, described as part of Tn*1546-*type transposons, generally carried on plasmids and thus effectively disseminated by HGT [6]. An acquisition of* vanA* plasmid by a strain of* E. faecium* representing hospital-adapted lineage may result in a spread of VR*Efm*, first colonizing patients and then causing symptomatic infections. Therefore, both characterization of the Tn*1546* structure and its linkage to particular plasmid groups is crucial for understanding of VRE dissemination in hospital environments. Several studies have shown the presence of various Tn*1546* types on Inc18, pRUM-like, pMG1-like, and pLG1 plasmids [7–13]; however, our knowledge of* vanA* plasmids and their epidemiology is still far from being satisfactory and the common presence of plasmids with Tn*1546,* belonging to unknown replicon types, has been shown [10, 14].

The aim of this study was to characterize* E. faecium* VanA isolates from the outbreaks that concomitantly took place in hospital wards of two neighboring medical centers, The Institute of Oncology (IO) and The Institute of Hematology and Transfusion Medicine in Warsaw (IH). The investigation focused on the clonal relationships among isolates as well as analysis of the Tn*1546* transposon structure and colocalization of* vanA* with other plasmid genes in order to elucidate the role of particular MGE during a VR*Efm* outbreak in hospital settings.

## 2. Materials and Methods

### 2.1. Outbreak Description, Bacterial Isolates, and Susceptibility Testing

Forty-four vancomycin-resistant* E. faecium* outbreak isolates were collected between February and June 2009 in two neighboring hospitals in Warsaw, The Institute of Oncology (IO) and The Institute of Hematology and Transfusion Medicine (IH), 776- and 198-bed hospitals, respectively. First VR*Efm* was isolated from stool of 46-year-old patient on 4th February at the Gastroenterology Clinic of IO. Until the end of February, eight more cases were reported, in majority from the Clinic of Lymphatic System Cancers of IO. From the 31st March till the 18th of April, 18 VR*Efm* were isolated, mainly from patients of this clinic (16 cases) and from two patients of the Gastroenterology Clinic. Simultaneously, VR*Efm* cases were reported in IH wards, with the first two isolations on the 5th February from rectum and stool of the Hematology Ward patient and a patient from the ICU, respectively. One more isolate was obtained 10 days later in the Surgery Ward and till the end of June, 14 other VR*Efm* cases were reported in the Hematology Ward of IH. Altogether, the outbreaks affected 42 patients, including 27 patients of IO (27 stool isolates) and 15 patients of IH (13 stool, 1 urine, 3 blood isolates). Antimicrobial susceptibility of collected isolates was determined using the Etest method (bioMérieux, Marcy l'Etoile, France) for glycopeptide susceptibility testing and broth microdilution method for other antimicrobials. The results were interpreted following the breakpoints of the European Committee on Antimicrobial Susceptibility Testing (EUCAST) [15]; for chloramphenicol, erythromycin, ciprofloxacin, and tetracycline the Clinical and Laboratory Standards Institute (CLSI) [16] breakpoints were applied, and in the case of kanamycin and clindamycin, the breakpoints proposed by the Société Française de Microbiologie (SFM) [17] were used. The* Enterococcus faecalis* strain ATCC29212 was used for quality control purposes during testing.* E. faecium* BM4147 was used as a control VanA strain in this study.

### 2.2. DNA Isolation and Genotyping of Isolates

Total DNA of isolates was extracted using Genomic DNA Prep Plus kit (A&A Biotechnology, Gdansk, Poland), following the manufacturer's instructions. Additionally, as the above method may result in a low yield of small plasmids, plasmid DNA was isolated using the alkaline lysis method [18]. Pulsed-field gel electrophoresis (PFGE) was performed according to de Lancastre et al. [19] for agarose plugs preparation, followed by the procedure of Clark et al. [20] for total genomic DNA purification. Purified DNA in plugs was digested with the* Sma*I restriction enzyme (Fermentas, Vilnius, Lithuania). Electrophoresis was performed at 14°C for 22 h with a pulse time of 1–30 s at 6 V/cm² in 0.5x TBE buffer and the results were interpreted according to criteria proposed by Tenover et al. [21]. The Bionumeric software (Applied Maths, Kortrijk, Belgium) was used to analyze the similarity of PFGE-banding patterns, with an unweighted pair group method with arithmetic average (UPGMA) algorithm and Dice coefficient. Multilocus variable-number tandem repeat (VNTR) analysis (MLVA) was performed as described by Top et al. [22] with modifications given on the website (http://www.mlva.umcutrecht.nl). Multilocus sequence typing (MLST) was performed as described previously [23]. Allele numbers and sequence types (STs) were assigned using* E. faecium* MLST database (http://efaecium.mlst.net/; 16th December 2013, date last accessed). PCR detection of IS*16* was performed as described by Werner et al. [24]. The Simpson index and Wallace index were calculated using the online tool available at http://darwin.phyloviz.net/ComparingPartitions/ (14th January 2014, date last accessed).

### 2.3. Detection of Virulence Genes, Antimicrobial Resistance Determinants, and Plasmid Functional Modules by PCR 

Enterococcal virulence genes *hyl*_Efm_, *esp*_Efm_,* gel*,* asa,* and* cyl* were screened as described by Vankerckhoven et al. [25]. Genes representing four* E. faecium* pilus gene clusters (PGC) were detected by the amplification of representative components of particular PGC, namely,* fms21/pilA* (PGC-1),* fms17* (PGC-2),* fms5* (PGC-3), and* fms19* (PGC-4) [26]. Antimicrobial resistance determinants were investigated using primers and conditions described by others:* vanA* [20],* cat* [27],* erm(B)* [28],* tet(M)* [29],* tet(O)* [30],* aad6* [31],* aac(6*′*)-Ie-aph(2*′′*)-Ia, aph(2*′′*)-Ib, aph(2*′′*)-Ic, aph(2*′′*)-Id, aph(3*′*)-IIIa*, and* ant(4*′*)-Ia* [32]. The presence of the* vanA* gene for 13 isolates from the IO was established in our previous study [33]. Detection of 19* rep* families and the unique *rep*_pMG1_ gene was performed according to Jensen at al. [34]. PCR for the *rep*_pLG1_ [9] was performed with primers designed previously [35]. The presence of plasmid addiction systems, relaxase genes, and the* intA* integrase gene of integrative and conjugative element ICE*Efm1* was also verified by PCR [36–38].

### 2.4. Plasmid Profiling, Hybridization Analyses, and Tn1546 Typing

DNA in agarose plugs obtained as described above was treated with 14U of S1 nuclease (Takara Bio, Japan) for 15 minutes at 37°C and separated by PFGE at 14°C for 22 h with pulse time 5–35 s at 6 V/cm² in 0.5x TBE buffer [39]. This method allows visualization and determination of the number and size of plasmids larger than approximately 30 kb. After electrophoresis, DNA was blotted onto the Hybond-N+ membrane (GE Healthcare, Buckinghamshire, UK) by capillary transfer. Probe labeling and signal detection for PFGE-S1 membranes were carried out using the Amersham ECL Random-Prime Labeling and Detection System (GE Healthcare), according to the manufacturer's protocol.

The 4.4 kb fragment of the* vanRSHAX* operon was amplified using Expand Long Template System (Roche Diagnostics GmbH, Mannheim, Germany) according to Palepou et al. [40] with the following amplification conditions: 94°C for 2 min; 10 cycles of 94°C for 10 s, 56°C for 30 s, and 68°C for 4 min; 20 cycles of 94°C for 10 s, 56°C for 30 s, and 68°C for 4 min (with the elongation time increased by 20 s/cycle); and 68°C for 7 min. L-PCR amplicons were analyzed by restriction fragment length polymorphism (RFLP) with* Dde*I (New England Biolabs, UK). The whole Tn*1546* transposon was investigated by PCR mapping and sequencing (Table 1 and references therein).

### 2.5. Conjugation Experiments

Conjugation transfer of vancomycin resistance was examined by cross-streak mating procedure with* E. faecium* strain 64/3 resistant to rifampin and fusidic acid as recipient. Fresh colonies of donors were cross-streaked with recipient on BHI-Agar plates and incubated overnight at 37°C. Bacterial cells from the streak crossing area were then incubated overnight in 37°C on selective media. Transconjugants were then confirmed by MLVA. For isolates negative for conjugation in this assay, a technique specific for bacteria with low frequency of transfer was used [44].

## 3. Results

### 3.1. Antibiotic Resistance Phenotypes, Antimicrobial Resistance Determinants, and Virulence Genes

**Figure 1:**
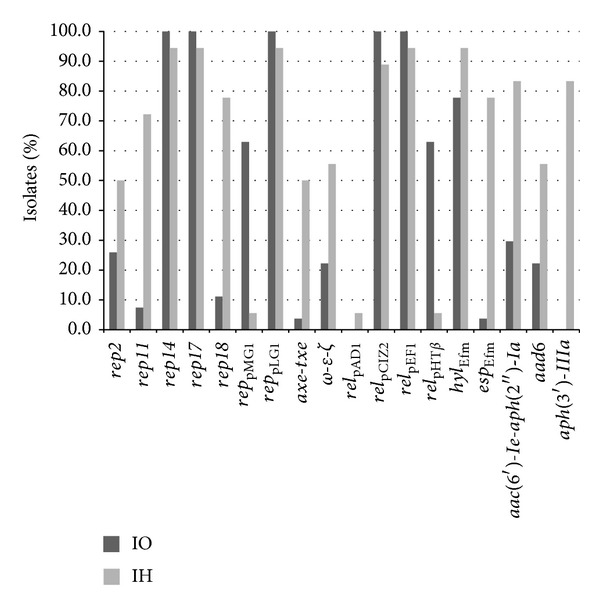
Distribution (% of isolates) of plasmid-specific genes, selected virulence genes, and resistance determinants among isolates from outbreak at the Institute of Oncology and Institute of Hematology, Warsaw.

All analyzed isolates were resistant to vancomycin and teicoplanin and exhibited the presence of* vanA* determinant (Table 2). Additionally, all of them were penicillin-, ampicillin-, ciprofloxacin-, clindamycin-, and erythromycin-resistant. The vast majority of isolates from both IO and IH showed resistance to rifampin. High-level resistance to gentamicin (HLGR), kanamycin (HLKR), and streptomycin (HLSR) was more prevalent among IH isolates, which were particularly enriched in aminoglycoside resistance genes* aac(6*′*)-Ie-aph(2*′′*)-Ia, aph(3*′*)-IIIa, *and* aad6 *(Figure 1). The* aph(2*′′*)-Ib* gene occurred in nine isolates and three other tested genes, coding for aminoglycoside resistance; that is,* aph(2*′′*)-Ic, aph(2*′′*)-Id*, and* ant(4*′*)-Ia* were not detected. Isolates from both groups commonly carried* erm(B)* and* tet(M)* genes. Resistance and intermediate susceptibility to tetracycline was typical for 61% and 18% of isolates, respectively. Intermediate susceptibility to chloramphenicol and quinupristin-dalfopristin was shown for 51% and 29% of isolates, respectively. All isolates were susceptible to linezolid and tigecycline.

Among virulence determinants studied, the *hyl*_Efm_ gene was prevalent in both outbreaks, while the *esp*_Efm_ gene was present mainly in IH (Table 2 and Figure 1). All *esp*_Efm_-positive isolates harbored the* intA* integrase gene. PGC genes* fms21* (PGC-1),* fms5* (PGC-3), and* fms19* (PGC-4) commonly occurred in the whole studied collection, while the* fms17* (PGC-2) was more prevalent among IH than IO isolates. Genes* gel*,* asa,* and* cyl* were not detected.

### 3.2. Clonal Relationships among Isolates

**Figure 2:**
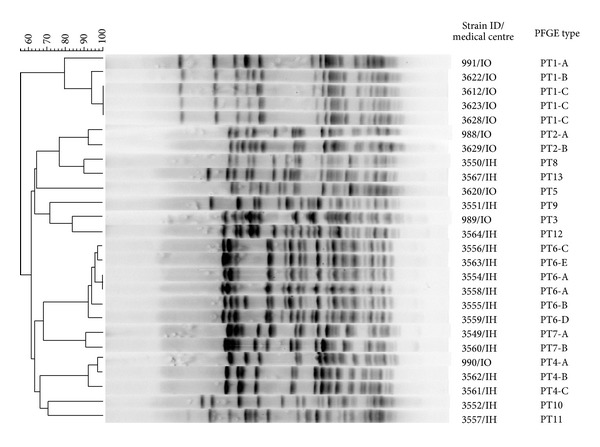
PFGE-based dendrogram of selected isolates from outbreaks in IO and IH, representing all PTs. Normalization performed by the use of reference Lambda Ladder PFG Marker (New England BioLabs, UK). The phylogenetic tree was constructed by the use of Dice coefficient (optimization, 0.5%; band tolerance, 1.3%) and UPGMA clustering.

The clonal structure of outbreak isolates was evaluated with the use of three typing methods (Table 2 and Figure 2). PFGE analysis and MLVA were performed for the whole set of isolates, yielding altogether 13 PFGE types (PTs) and eight MLVA types (MTs). Two PTs, PT1 and PT6, were further diversified into three and five subtypes, respectively. A single novel MT296 was detected for the isolate recovered from blood in the IH. Generally, the IH isolates showed higher diversity of PTs and MTs, compared to the isolates obtained from the IO. For both hospitals, the predominance of specific PTs and MTs was observed, namely, PT1/MT3 and PT5/MT10 among the IO isolates and PT6/MT159 in the IH. Isolates with MT1/PT4 were observed in the two institutions (a single isolate in both IO and IH). In the case of two patients, two VR*Efm* isolates with different PFGE and MLVA types were obtained from different body sites (rectum, stool, and blood). The comparison of MLVA and PFGE typing results showed a good correlation of both methods (Wallace indices: PT/MT 0.994, MT/PT 0.887) and a higher discriminatory power of PFGE over MLVA (Simpson's indices 81.7 and 79.5, resp.). Further MLST analysis for 20 representatives of different PTs and MTs yielded six sequence types (STs), all belonging to lineages: 17 (STs 17, 202), 18 (STs 18, 262), and 78 (STs 78, 192) of meroclone CC17. The most common ST18 occurred in both hospitals, however, in association with various PTs/MTs; this ST was also characteristic for two MT1/PT4 isolates mentioned above.

### 3.3. Tn1546 Structures and Transferability of Vancomycin Resistance

All isolates exhibited the presence of 4.4 kb L-PCR product containing the* vanRSHAX* operon and showed that the* DdeI* restriction pattern is identical to the* E. faecium* BM4147 control VanA strain. Further PCR mapping and sequencing showed the presence of IS*Ef1,* inserted at the position 9147 nt of Tn*1546* (numbering according to the GenBank sequence M97297), that is, within the* vanX-vanY* intergenic region. The 5′GACTGAAA duplication was observed at the insertion site. IS*Ef1* was present in all but two isolates with the prototype Tn*1546.* One of the isolates was derived from IO and the other from IH, and each of them showed a unique PFGE type, PT3 and PT10, respectively (Table 2). Similarly, all isolates, except for the two mentioned above, exhibited the G7747C point mutation of Tn*1546* located within the* vanA* gene, resulting in the amino acid substitution V257F. Both isolates with the prototype Tn*1546* showed higher teicoplanin MIC values compared to the isolates with Tn*1546:* IS*Ef1.*

Conjugation experiments were performed for all 44 isolates and 34 of them were able to transfer vancomycin resistance to the* E. faecium* 64/3 recipient. All donors produced transconjugants with cross-streak mating except for a single isolate, which required use of the method designed for strains with low-level conjugation frequencies [44]. Susceptibility testing of transconjugants (a single transconjugant for each donor) showed a concomitant transfer of erythromycin resistance in 32 cases. One of these transconjugants showed also HLGR phenotype and one was additionally resistant to tetracycline.

### 3.4. Diversity of Plasmid-Associated Gene Content

PCR screening revealed the presence of plasmid replication genes of the *rep*14_pRI_ and *rep*17_pRUM_ families as well as *rep*_pLG1_ in all isolates. Four other* rep* groups, *rep*2_pRE25_ (*n* = 16), *rep*11_pEF1071_ (*n* = 15), *rep*18_pEF418_ (*n* = 19), and *rep*_pMG1_ (*n* = 18) were also detected. First three of them were characteristic mainly for the IH outbreak, while *rep*_pMG1_ occurred mainly in isolates from IO (Figure 1). The number of* rep* genes per isolate varied from three to seven, and the average number of plasmid* rep* genes per isolate was 4.50; however, this value was lower for IO (4.07) compared to IH (5.18). Analysis of distribution of relaxase genes revealed the common presence of two relaxases, *rel*_pCIZ2_ and *rel*_pEF1_, while *rel*_pHT*β*_ was predominantly detected in IO and the distribution of this gene was completely concordant with the presence of *rep*_pMG1_. Additionally, one IH isolate had the *rel*_pAD1_ gene. Screening for plasmid toxin-antitoxin systems (TA) resulted in the detection of* axe-txe* and **ω*-*ε*-*ζ*,* while other TA genes, including* ccd, higBA, mazEF, par, parDE, phd-doc, relBE, *and* vagCD* were absent in the studied group. All but one  *ω* *-*ε*-*ζ**-positive isolates were also *rep*2_pRE25_-positive and only one *rep*2_pRE25_-positive lacked the  *ω* *-*ε*-*ζ** gene.

### 3.5. Colocalization of vanA Determinant and Other Plasmid Genes

**Figure 3:**
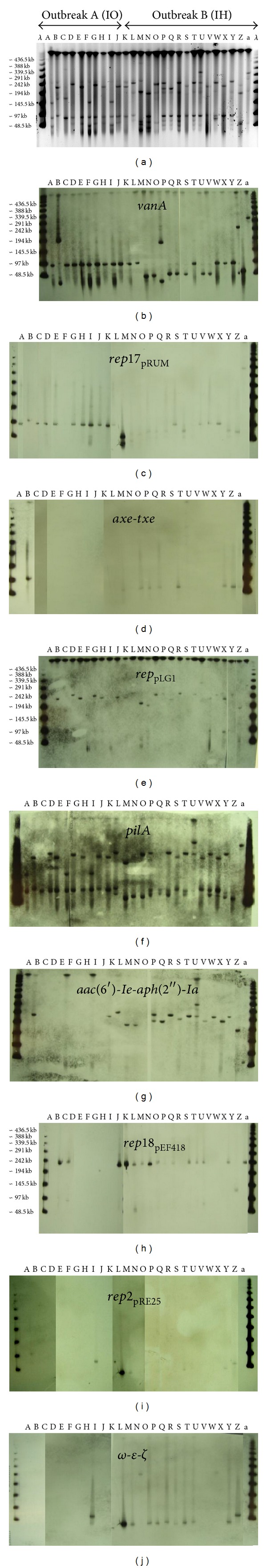
PFGE of S1-digested total DNA of selected 27 VR*Efm* isolates, visualized by ethidium bromide staining (a) and subjected to Southern hybridization with the following probes:* vanA* (b), *rep*17_pRUM_ (c),* axe-txe* (d), *rep*_pLG1_ (e),* pilA* (f),* aac(6*′*)-Ie-aph(2*′′*)-Ia* (g), *rep*18_pEF418_ (h), *rep*2_pRE25_ (i), and  *ω* *-*ε*-*ζ** (j). Lanes A–a, isolates designation as described in Table 3.

Twenty-seven selected isolates (11 from IO and 16 from IH) of various clonal types, as defined by MLVA, MLST, and PFGE, were subjected to PFGE-S1 analysis, followed by Southern blot hybridization (Figure 3 and Table 3) with probes specific for genes detected earlier by PCR, such as* vanA*, seven* rep* genes (*rep*2_pRE25_, *rep*11_pEF1071_, *rep*14_pRI1_, *rep*17_pRUM_, *rep*18_pEF418_, *rep*_pLG1_, and *rep*_pMG1_), genes of two plasmid TA systems (*ω* *-*ε*-*ζ** and* axe-txe*), and three other plasmid-associated genes (*pilA*, *hyl*_Efm_, and* aac(6*′*)-Ie-aph(2*′′*)-Ia*). Altogether, 122 plasmid bands were visualized in PFGE-S1, with 56 megaplasmids bands greater than 100 kb. The average number of plasmid bands in PFGE-S1 analysis was 4.35, with very similar values for both IO and IH outbreaks. Thirty plasmid bands hybridized with the* vanA* probe; that is, three of the analyzed isolates carried two* vanA* plasmids. The majority of* vanA* plasmids were <30–100 kb in size; additionally, four megaplasmids (170, 200, 240, and 315 kb) were associated with the* vanA* determinant. Among* vanA* plasmids, 24 were *rep*17_pRUM_ replicons, mostly of 50 kb and 100 kb. The 100 kb plasmid was present in 15 isolates of various clonal backgrounds in both IH and IO and most of these isolates easily produced transconjugants. Moreover, the first observed VR*Efm* isolates in both IH and IO carried such plasmids but in different clonal backgrounds. The 50 kb plasmid was associated exclusively with MT159 isolates, which differed, however, in their PFGE patterns. Six such isolates occurred exclusively in IH and all of them were deficient in conjugation. Five of* vanA*-*rep*17_pRUM_ plasmids of various sizes hybridized also with other* rep* genes, such as *rep*_pLG1_ (two 100 kb plasmids and one 315 kb plasmid), *rep*18_pEF418_ (one 100 kb plasmid), and both *rep*2_pRE25_ and *rep*18_pEF418_ (a 40 kb plasmid). Two isolates of MT1/PT4/ST18 from IO and IH both had 100 kb *rep*17_pRUM_ plasmids but they differed in the content of other plasmids (Table 3, isolates labeled C and X); moreover, 100 kb plasmids from IO isolate additionally carried *rep*18_pEF418_. Among the remaining plasmids, other than *rep*17_pRUM_ replicons,* vanA* plasmids, a single 70 kb plasmid had *rep*2_pRE25_ and a 240 kb megaplasmid hybridized with *rep*_pLG1_ and *rep*18_pEF418_. Considering other tested genes, the* pilA* gene was associated with 18* vanA* plasmids, including all the 100 kb plasmids with *rep*17_pRUM_; genes of the  *ω* *-*ε*-*ζ** TA system were present on a single 40 kb plasmid with *rep*2_pRE25_, *rep*17_pRUM_, and *rep*18_pEF418_ and on a 70 kb plasmid carrying *rep*2_pRE25_. The 240 kb* vanA* megaplasmid carried also the* aac(6*′*)-Ie-aph(2*′′*)-Ia* resistance gene. Each of the two isolates with the prototype Tn*1546* carried two* vanA* plasmids (<30 kb in both isolates and megaplasmids of 170 and 200 kb) that did not hybridize with any of probes used in this study. In summary, almost all IO isolates showed the presence of 100 kb* vanA* plasmids with *rep*17_pRUM_ and* pilA* genes, while in IH the diversity of* vanA* plasmids was higher, with pRUM-like replicons of both 50 kb and 100 kb Information concerning other than* vanA* plasmids of* E. faecium* that was obtained during the study is summarized in Table 3.

## 4. Discussion

This study provides the molecular characteristics of VR*Efm* outbreak isolates with the special focus on the role of MGE, such as Tn*1546-*type transposons and* vanA* plasmids, acting as mediators of vancomycin resistance transfer. The investigated group of isolates originated from two hospitals, The Institute of Oncology and The Institute of Hematology and Transfusion Medicine in Warsaw, where two VR*Efm* outbreaks occurred concomitantly. Immunocompromised patients of oncological and hematological wards are known to be of special risk for VRE colonization and infection [45]. Such susceptibility was especially evident during the outbreak in the IH, where three bloodstream infections caused by VRE were reported. The proximity of the two hospitals in the city and the simultaneous emergence of both outbreaks, which lasted for a few months, suggested the possibility of VRE transmission between the two institutions, although the investigation of available medical documentation, done independently in IO and IH, revealed no obvious routes, such as patient transfer between the two hospitals or from the same third hospital or utilization of common diagnostic equipment just before or during the outbreak period. The involvement of hospital personnel in VR*Efm* transfer was also excluded.

Molecular typing methods, such as PFGE analysis and MLVA, have shown a divergent clonal structure of isolates. Such a situation is typical for VanA hospital outbreaks [46–49]. MLST performed for representative isolates included all of them into the hospital-associated lineages 17, 18, and 78, formerly described as CC17 complex [3]. Isolates belonging to this meroclone display common features such as ampicillin and ciprofloxacin resistance, the prevalence of IS*16*, *esp*_Efm_, and *hyl*_Efm_, and enrichment in microbial surface components recognizing adhesive matrix molecules (MSCRAMMs), including pili genes [26, 50]. However, isolates from each institution showed some specific features, such as predominance of certain PTs/MTs and differences in distribution of virulence, resistance, and plasmid-specific genes. This observation would suggest the existence of two separate endemic subpopulations without much exchange of strains prior to the introduction of* vanA-*carrying MGE.

VanA phenotype in both outbreaks was associated, in the vast majority of cases, with an acquisition of the same specific variant of Tn*1546* transposon with IS*Ef1* and a mutation in the* vanA* gene. Tn*1546-*type transposons show significant variability due to point mutations, deletions, and presence of various ISs [41, 42, 47, 51], and thus analysis of transposon structure provides valuable epidemiological information for investigation of VRE outbreaks. The IS*Ef1* insertion in the* vanX-vanY* intergenic region was described previously for hospital VR*Efm* in Portugal and Germany [47, 51], and the mutation in the* vanA* gene was not reported before. The presence of the same type of Tn*1546* in both outbreaks provides a strong indication of either a common source or transmission of VR*Efm* between the two hospitals.

Hybridization studies on representative isolates revealed the presence of a 100 kb plasmid carrying the* vanA* determinant as well as* rep17,* typical for pRUM plasmid and the* pilA* gene in several isolates from both institutions. Most of these isolates readily produced transconjugants, suggesting that this plasmid might play the principal role in the outbreak. The observed concomitant transfer of erythromycin resistance is in agreement with the colocalization of the* ermB* gene on pRUM [52]. Other, frequently encountered* vanA* plasmid was 50 kb in size and also represented the *rep*17_pRUM_ replicon; however, it lacked* pilA* and all isolates with this plasmid were negative in conjugation. The 50 kb plasmid was exclusively associated with isolates of MT159/ST78 and observed solely in IH. The recently emerged lineage 78 of the hospital-adapted* E. faecium* shows increased epidemic properties and plays an important role in HAIs [53]. Thus, strains of this lineage, harboring the 50 kb nonconjugative* vanA* plasmid, were likely to be spreading by efficient clonal dissemination during the outbreak in IH. Association of* van* determinants with pRUM-type plasmids was described also by others [7, 10, 54]. Both 100 kb and 50 kb plasmids lacked the* axe-txe* TA system genes, typical for pRUM [10, 52], suggesting the possible common origin of these two plasmids. Further studies, based on whole plasmid sequencing, are indispensable to elucidate the possible evolution of these plasmids during the outbreak. Although 100 kb plasmids were found in two isolates of the same clonal characteristics from IO and IH (Table 3, isolates C and X), differences in the plasmid content do not allow us to indicate these isolates as a direct epidemiological link between two hospitals. In a few cases, the* vanA* gene was associated with other replicons, typical for pLG1, pRE25, and pEF418. Such* vanA* plasmids were observed also in other studies [7, 10]. The prevalent distribution of *rep*_pLG1_ as well as its predominant presence on plasmids over 200 kb in size is also in agreement with earlier studies which showed that all VR*Efm* megaplasmids with the defined replicon type were always pLG1-like [7, 8]. The association of VanA determinants with various plasmids during one outbreak may be caused by the Tn*1546* transposition among plasmids and/or plasmid recombination. The latter process may yield plasmids with more than a single* rep* gene, which was also observed in the current study, both for* vanA-* and other plasmids. The role of plasmid mosaics in the dissemination of Tn*1546* among VRE was emphasized recently by Freitas et al. [7]. Finally, for some* vanA* plasmids and other plasmids the replicon types could not be established (13% and 34% of observed plasmids, resp.), indicating that the pool of* E. faecium* plasmids remains only partly explored [10] and that there is the need for further studies of these epidemiologically important elements.

## 5. Conclusions

Molecular analysis of VanA VR*Efm* outbreaks revealed that Tn*1546*::IS*Ef1* elements associated with pRUM-like plasmids were the key mediators of vancomycin-resistant* E. faecium* dissemination among the investigated group of oncological/hematological patients. Horizontal gene transfer of the whole* vanA* plasmids and/or Tn*1546* transposons in endemic populations of nosocomial* E. faecium* is suggested as the potential way of VanA phenotype spread in the analyzed outbreaks. The enrichment in different plasmid-associated genes, antimicrobial resistance, and potential virulence determinants in the investigated population emphasizes the impact of mobile genetic elements on the epidemiology and evolution of VR*Efm*.

## Figures and Tables

**Table 1:** Primers used in the analysis of Tn*1546 * transposon.

Primer pair	Primer names	Sequence (5′-3′)	Position in Tn*1546 *	Application in this study	Reference
1	vanRSHAX-1	AGACAAGTCTGAGATTGACCTTGCC	4141–4165	PCR	[40]
	vanRSHAX-2	ATATGCTTCAAACCCACTGTTTTCC	8565–8589	PCR	[40]
2	Tn*1546 *	GGAAAATGCGGATTTACAACGCTAAG	13–38	PCR	[40]
	ORF1-5	CACGTCCTGCCGACTATGATTATTT	1900–1876	PCR	[41]
3	ORF2-F	TCATTCCATTTCTGTATTTTCAATTT	3050–3086	PCR	[42]
	ORF2-R	GCCCATTAGCGGAATACAGA	3770–3751	PCR	[42]
4	ORF2-F2	ACTAATGTATCTAGGGCTTCA	3710–3731	PCR	[42]
	vanR-R	GCAATTTCATGTTCATCATCCA	4000–3979	PCR	[42]
5	vanS	AACGCTATTCCAAACTAGAA	4690–4710	PCR, sequencing	[41]
	vanS-R	GTCGGAAGCTCTACCCTAAA	5760–5741	PCR, sequencing	[41]
6	vanS1	ATTGTTCAGCATGGAGGGC	5700–5719	PCR, sequencing	[34]
	vanH2	GAGCATGGAATGCATCTGCC	6060–6041	PCR	[34]
7	vanA1	CATGAATAGAATAAAAGTTGCAATA	6978–7002	PCR	[20]
	vanX2	TTATTTAACGGGGAAATC	8600–8583	PCR, sequencing	[43]
8	vanX-F	ATGGGTATTTTCAGAAGTCCC	8580–8601	PCR, sequencing	[42]
	vanZ2	AATGGGTACGGTAAACGAGC	10555–10536	PCR	[34]
ORF1-4		GCATGTAGTGATGAAACACCTAGCTGC	960–987	sequencing	[41]
vanA2		CCCCTTTAACGCTAATACCATCAA	8007–7894	sequencing	[20]
vanY1		AGAGACGAACCATACCCCAA	9200–9181	sequencing	[42]
vanY2-R		AGTATGTGTTGATCCGGGAAAC	9900–9922	sequencing	this study

**Table 2:** Clonal relatedness, antimicrobial resistance profiles, and distribution of resistance and virulence determinants among IO and IH outbreak isolates.

Isolate	MT/PT/ST	MIC VAN (mg/L)	MIC TEI (mg/L)	Resistance phenotypes^a,b^	Resistance determinants^b,c^	Cotransferred resistance	Virulence genes	Pili genes^d^
Institute of oncology								
988	1/2-A/17	>256	32	RIF	*ermB *	ERY	—	*fms17, fms5, fms19 *
989*	159/3/78	>256	256	CHL, GEN, KAN, RIF	*aac(6′)-Ie-aph(2′′)-Ia, cat, tetM *	—	*esp, hyl *	*fms5, fms19 *
990	1/4-A/18	>256	32	RIF	*ermB, tetM *	ERY	*hyl *	*fms5, fms19 *
991	3/1-A/18	>256	32	RIF	*ermB, tetM *	ERY	*hyl *	*fms5, fms19 *
992	3/1-A/nd	>256	32	RIF	*ermB, tetM *	ERY	*hyl *	*fms5, fms19 *
993	3/1-A/nd	>256	32	RIF	*ermB, tetM *	ERY	*hyl *	*fms5, fms19 *
994	3/1-A/nd	>256	32	RIF	*ermB, tetM *	ERY	*hyl *	*fms5, fms19 *
995	3/1-A/nd	>256	24	RIF, TET	*ermB, tetM *	ERY	*hyl *	*fms5, fms19 *
996	3/1-A/nd	>256	32	RIF, TET	*ermB, tetM *	ERY	*hyl *	*fms5, fms19 *
3612	3/1-C/nd	>256	48	GEN, RIF, TET	*ermB, tetM *	ERY	*hyl *	*fms5, fms19 *
3613	3/1-C/nd	>256	32	RIF, TET	*ermB, tetM *	ERY	*hyl *	*fms5, fms19 *
3614	3/1-C/nd	>256	32	RIF, TET	*ermB, tetM *	ERY	*hyl *	*fms5, fms19 *
3615	3/1-C/nd	>256	32	RIF, TET	*ermB, tetM *	ERY	*hyl *	*fms5, fms19 *
3616	3/1-C/nd	>256	32	RIF, TET	*ermB, tetM *	ERY	*hyl *	*fms5, fms19 *
3617	3/1-C/nd	>256	32	RIF, TET	*ermB, tetM *	ERY	*hyl *	*fms5, fms19 *
3618	3/1-C/nd	>256	32	RIF, TET	*ermB, tetM *	ERY	*hyl *	*fms5, fms19 *
3620	10/5/262	>256	48	GEN, KAN, RIF, STR, TET	*aac(6′)-Ie-aph(2′′)-Ia, aad6, ermB, tetM *	ERY	*hyl *	*fms17, fms5, fms19 *
3621	3/1-C/nd	>256	32	RIF, TET	*ermB, tetM *	ERY	*hyl *	*fms5, fms19 *
3622	3/1-B/18	>256	32	RIF, TET	*ermB, tetM *	ERY	*hyl *	*fms5, fms19 *
3623	3/1-C/nd	>256	48	RIF, TET	*ermB, tetM *	ERY	*hyl *	*fms5, fms19 *
3624	10/5/262	>256	48	GEN, KAN, RIF, STR, TET	*aac(6′)-Ie-aph(2′′)-Ia, aad6, ermB, tetM *	no	—	*fms17, fms5, fms19 *
3625	10/5/nd	>256	48	GEN, KAN, RIF, STR, TET	*aac(6′)-Ie-aph(2′′)-Ia, aad6, ermB, tetM *	no	—	*fms17, fms5, fms19 *
3626	10/5/nd	>256	48	GEN, KAN, RIF, STR, TET	*aac(6′)-Ie-aph(2′′)-Ia, aad6, ermB, tetM *	no	—	*fms17, fms5, fms19 *
3627	10/5/nd	>256	48	GEN, KAN, RIF, STR, TET	*aac(6′)-Ie-aph(2′′)-Ia, aad6, ermB, tetM *	ERY	—	*fms17, fms5, fms19 *
3628	3/1-C/nd	>256	64	RIF, TET	*ermB, tetM *	ERY	*hyl *	*fms5, fms19 *
3629	7/2-B/18	>256	48	GEN, KAN, TET	*aac(6′)-Ie-aph(2′′)-Ia, ermB, tetM *	ERY	*hyl *	*fms17, fms5, fms19 *
3630	10/5/nd	>256	64	GEN, KAN, RIF, STR, TET	*aac(6′)-Ie-aph(2′′)-Ia, aad6, ermB, tetM *	ERY, TET	—	*fms17, fms5, fms19 *
**IO** (**N** = **27**)				**CHL** (**1**), **GEN** (**9**), **KAN** (**8**), **STR** (**6**), **TET** (**20**), **RIF** (**26**)	***cat*** (**1**), ***aac***(**6**′)***-Ie-aph***(**2**′′)***-Ia*** (**8**), ***aad6***, (**6**), ***ermB*** (**26**), ***tetM*** (**26**)	**ERY** (**23**)**, TET** (**1**)	***hyl*** (**21**), ***esp*** (**1**)	***fms17*** (**8**), ***fms5*** (**27**), ***fms19*** (**27**)

Institute of hematology								
3549	11/7-A/202	>256	32	GEN, KAN, RIF, TET	*aac(6′)-Ie-aph(2′′)-Ia, aph(2′′)-Ib, ermB, tetM *	ERY, GEN	*esp, hyl *	*fms17, fms5, fms19 *
3550^x^	7/8/18	>256	48	GEN, KAN, RIF, STR	*aac(6′)-Ie-aph(2′′)-Ia, aph(2′′)-Ib, aph(3′)-IIIa, aad6, ermB *	ERY	*esp, hyl *	*fms17, fms5, fms19 *
3551	159/9/78	>256	32	GEN, KAN, RIF, STR	*aac(6′)-Ie-aph(2′′)-Ia, aph(2′′)-Ib, aph(3′)IIIa, aad6, ermB *	ERY	*esp, hyl *	*fms17, fms19 *
3552*	7/10/18	>256	>256	KAN, RIF, STR	*aph(3′)-IIIa, aad6, ermB, tetM *	ERY	*esp, hyl *	*fms17, fms5, fms19 *
3553	11/7-A/nd	>256	32	GEN, KAN, RIF, TET	*aac(6′)-Ie-aph(2′′)-Ia, aph(2′′)Ib, aph(3′)IIIa, ermB, tetM *	ERY	*esp, hyl *	*fms17, fms5, fms19 *
3554	159/6-A/192	>256	48	GEN, KAN, RIF, STR	*aac(6′)-Ie-aph(2′′)-Ia, aph(2′′)-Ib, aph(3′)-IIIa, aad6, ermB *	no	*esp, hyl *	*fms17, fms5, fms19 *
3555	159/6-B/nd	>256	48	GEN, KAN, RIF, STR	*aac(6′)-Ie-aph(2′′)-Ia, aph(2′′)-Ib, aph(3′)-IIIa, aad6, ermB *	no	*esp, hyl *	*fms17, fms5, fms19 *
3556	159/6-C/nd	>256	32	GEN, KAN, STR	*aac(6′)-Ie-aph(2′′)-Ia, aph(3′)-IIIa, aad6, ermB *	no	*esp, hyl *	*fms17, fms5, fms19 *
3557^x#^	296/11/17	>256	48	KAN, RIF	*aph(3′)-IIIa, aad6, cat, ermB, tetM *	ERY	*esp, hyl *	*fms17, fms19 *
3558	159/6-A/nd	>256	48	GEN, KAN, PEN	*aac(6′)-Ie-aph(2′′)-Ia, aph(3′)-IIIa, aad6, ermB *	no	*esp, hyl *	*fms17, fms5, fms19 *
3559	159/6-D/nd	>256	48	GEN, KAN, PEN	*aac(6′)-Ie-aph(2′′)-Ia, aph(3′)-IIIa, aad6, ermB *	no	*esp, hyl *	*fms17, fms5*,
3560	1/7-B/18	>256	48	GEN, KAN, RIF, TET	*aac(6′)-Ie-aph(2′′)-Ia, aph(3′)-IIIa, ermB, tetM *	ERY	*hyl *	*fms5, fms19 *
3561	11/4-C/202	>256	32	GEN, KAN, RIF, TET	*aac(6′)-Ie-aph(2′′)-Ia, aph(2′′)-Ib, aph(3′)-IIIa, ermB, tetM *	ERY	*esp, hyl *	*fms17, fms5*,
3562^#^	1/4-B/18	>256	32	GEN, KAN, RIF, TET	*aac(6′)-Ie-aph(2′′)-Ia, aph(3′)-IIIa, ermB, tetM *	no	*hyl *	*fms5, fms19 *
3563	159/6-E/192	>256	48	GEN, KAN, RIF	*aac(6′)-Ie-aph(2′′)-Ia, aph(2′′)-Ib, aph(3′)-IIIa, aad6, ermB *	no	*esp, hyl *	*fms17, fms5, fms19 *
3564^y^	7/12/18	>256	48	GEN, KAN, RIF, TET	*aac(6′)-Ie-aph(2′′)-Ia, aph(2′′)-Ib, aph(3′)-IIIa, ermB, tetM *	ERY	*esp, hyl *	*fms17, fms5, fms19 *
3567^y#^	144/13/18	>256	48	GEN, KAN, RIF, TET	*aac(6′)-Ie-aph(2′′)-Ia, aph(2′′)-Ib, ermB *	ERY	*hyl *	*fms17, fms5, fms19 *
**IH** (**N** = **17)**				**GEN** (**15**), **KAN** (**17**), **STR (6)**, **TET** (**7**), **RIF** (**15**)	***cat*** (**1**), ***aac***(**6**′)***-Ie-aph***(**2**′′)***-Ia*** (**15**), ***aph***(**2**′′)***-Ib*** (**10**), ***aph***(**3**′)***-IIIa*** (**15**), ***aad6*** (**10**), ***ermB*** (**17**), ***tetM*** (**8**)	**ERY** (**11**), **GEN** (**1**)	***hyl*** (**17**), ***esp* (14**)	***fms17*** (**15**), ***fms5*** (**15**), ***fms19*** (**15**)

**IO and IH** (**N** = **44**)				**CHL** (**1**), **GEN** (**24**), **KAN** (**25**), **STR** (**12**), **TET** (**27**), **RIF** (**41**)	*** cat*** (**2**), ***aac***(**6**′)***-Ie-aph***(**2**′′)***-Ia*** (**23**), ***aph***(**2**′′)***-Ib*** (**10**), ***aph***(**3**′)***-IIIa*** (**15**), ***aad6*** (**16**), ***ermB*** (**43**), ***tetM*** (**34**)	**ERY** (**34**), **TET** (**1**), **GEN** (**1**)	***hyl*** (**38**), ***esp*** (**15**)	***fms17*** (**23**), ***fms5*** (**42**), ***fms19*** (**42**)

nd: not determined; no: no transconjugants obtained; two isolates with the prototype Tn*1546* marked with an asterisk; ^#^isolates from blood; ^a^all isolates resistant to vancomycin, teicoplanin, penicillin, ampicillin, ciprofloxacin, erythromycin, and clindamycin; ^b^five isolates with *tet(M) *showed intermediate susceptibility to tetracycline and one isolate with *tet(M) *was susceptible to this compound, one tetracycline-resistant isolate (3567) was negative for the determinants tested; for four isolates with *aad*6 the MIC values for streptomycin increased (512 mg/L); one isolate with *cat *showed an intermediate resistance to chloramphenicol; ^c^all isolates were positive for the *vanA *gene; ^d^all isolates positive for the *pilA* gene; ^x,y^isolates from the same patients “X” and “Y”; RIF: rifampin; CHL: choramphenicol; GEN: gentamicin (HLGR); STR: streptomycin (HLSR); KAN: kanamycin (HLKR); TET: tetracycline. For the summarized results, the number of isolates is given in brackets.

**Table 3:** Plasmid profiles and colocalization of particular genes on *vanA* and other plasmids among selected 27 VR*Efm* isolates.

Letter code	Isolate	MT/PT/ST	*vanA* plasmids in kb (hybridizing probes)	Other plasmids in kb (hybridizing probes)
IO (*N* = 11)				
A	988^#^	1/2-A/17	100 (*rep17*, *pilA*)	<30, 150 (*pilA*), 180, 270
B	989^∗#^	159/3/78	<30, 200	110 (*rep2, rep17, pilA, axe-txe*), 175, 230 (*rep18, * *rep*_*pLG*1_ *, hyl, aac (6*′*)-Ie-aph (2*′′*)-Ia, pilA*), 310, >400
C	990^#^	1/4-A/18	100 (*rep17*, *rep18*, *pilA*)	175, 230 (*rep18*), 280, 340
D	991^#^	3/1-A/18	100 (rep17, pilA)	65 (*rep*_*pMG*1_), 240 (*rep*_*pLG*1_ *, hyl, pilA*), 295
E	3612^#^	3/1-C/nd	100 (*rep17, pilA*)	70 (*rep*_*pMG*1_), 235 (*rep*_*pLG*1_ *, hyl, pilA*), 290
F	3620^#^	10/5/262	100 (*rep17, pilA*)	65, 150 (*pilA*), 290
G	3622^#^	3/1-B/18	100 (*rep17, pilA*)	70 (*rep*_*pMG*1_), 240 (*rep*_*pLG*1_ *, hyl, pilA*), 290
H	3623^#^	3/1-C/nd	100 (*rep17, pilA*)	70 (*rep*_*pMG*1_), 235 (*rep*_*pLG*1_ *, hyl, pilA*), 290
I	3624	10/5/262	100 (*rep17, pilA*)	40, 65 (*rep2, *ω*-*ε*-*ζ**), 150 (*pilA*), 290
J	3628^#^	3/1-C/nd	100 (*rep17, pilA*)	70 (*rep*_*pMG*1_), 235 (*rep*_*pLG*1_ *, hyl, pilA*), 290
K	3629^#^	7/2-B/18	100 (*rep17, pilA*)	235 (*rep18, * *rep*_*pLG*1_ *, hyl, aac (6*′*)-Ie-aph (2*′′*)-Ia, pilA*), 270

IH (*N* = 16)				
L	3549^#^	11/7-A/202	100 (*rep17, pilA*)	70 (*rep*_*pMG*1_ *, pilA*), 240 (*rep18, * *rep*_*pLG*1_ *, aac (6*′*)-Ie-aph (2*′′*)-Ia, pilA*), 310
M	3550^x#^	7/8/18	45 (*rep2, rep17, rep18, *ω*-*ε*-*ζ**)	60 (*rep17, pilA*), 70, 195 (*rep*_*pLG*1_ *, aac (6*′*)-Ie-aph (2*′′*)-Ia, pilA*), 230 (*rep18*), 315
N	3551^#^	159/9/78	45 (*rep17*)	40 (*rep2, *ω*-*ε*-*ζ**), 70 (*rep17, pilA, axe-txe*), 100, 190 (*rep*_*pLG*1_ *, aac (6*′*)-Ie-aph (2*′′*)-Ia)*, 240 (*rep18*), 290
O	3552^∗#^	7/10/18	<30, 170	65 (*rep17, pilA*), 240 (*rep18, * *rep*_*pLG*1_ *, pilA, *ω*-*ε*-*ζ**)
P	3554	159/6-A/192	50 (*rep17*)	40 (*rep2, *ω*-*ε*-*ζ**), 75 (*rep17, pilA, axe-txe*), 100 (*pilA*), 220 (*aac (6*′*)-Ie-aph (2*′′*)-Ia)*, 240 (*rep18, * *rep*_*pLG*1_ *, aac (6*′*)-Ie-aph (2*′′*)-Ia, pilA*)
Q	3555	159/6-B/nd	50 (*rep17*)	40 (*ω* *-*ε*-*ζ**), 75 (*rep17, pilA, axe-txe*), 100 (*pilA*), 220 (*rep*_*pLG*1_ *, aac (6*′*)-Ie-aph (2*′′*)-Ia)*, 240 (*rep18*)
R	3556	159/6-C/nd	50 (*rep17*)	40 (*ω* *-*ε*-*ζ**), 75 (*rep17, pilA, axe-txe*), 100 (*pilA*), 240 (*rep18, * *rep*_*pLG*1_ *, aac (6*′*)-Ie-aph (2*′′*)-Ia, pilA*)
S	3557^x#^	296/11/17	100 (*rep17, pilA*)	40 (*ω* *-*ε*-*ζ**), 235 (*pilA*)
T	3558	159/6-A/nd	50 (*rep17*)	40 (*rep2, *ω*-*ε*-*ζ**), 75 (*rep17, pilA, axe-txe*), 100 (*pilA*), 240 (*rep18, * *rep*_*pLG*1_ *, aac (6*′*)-Ie-aph (2*′′*)-Ia, pilA*)
U	3559	159/6-D/nd	50 (*rep17*)	40 (*ω* *-*ε*-*ζ**), 75 (*rep17*), 100 (*rep17*), 240 (*rep18, aac (6*′*)-Ie-aph (2*′′*)-Ia, pilA*), 330 (*rep*_*pLG*1_ *, axe-txe, aac (6*′*)-Ie-aph (2*′′*)-Ia, pilA*)
V	3560^#^	1/7-B/18	100 (*rep17, * *rep*_*pLG*1_ *, pilA*)	85 (*pilA*), 235 (*rep18, * *rep*_*pLG*1_ *, aac (6*′*)-Ie-aph (2*′′*)-Ia, pilA*)
W	3561^#^	11/4-C/202	100 (*rep17, * *rep*_*pLG*1_ *, pilA*)	240 (*rep*_*pLG*1_ *, aac (6*′*)-Ie-aph (2*′′*)-Ia, pilA*)
X	3562	1/4-B/18	100 (*rep17, pilA*)	240 (*rep*_*pLG*1_ *, aac (6*′*)-Ie-aph (2*′′*)-Ia, pilA*)
Y	3563	159/6-E/192	50 (*rep17*), 240 (*rep18, * *rep*_*pLG*1_ *, pilA, aac(6*′*)-Ie-aph(2*′′*)-Ia*)	40 (*rep2, *ω*-*ε*-*ζ**), 75 (*rep17, pilA, axe-txe*), 100 (*pilA*), 240 (*rep18, * *rep*_*pLG*1_ *, aac (6*′*)-Ie-aph (2*′′*)-Ia, pilA*)
Z	3564^y#^	7/12/18	70 (*rep2, pilA,ω*-*ε*-*ζ*)	60 (*rep*_*pLG*1_), 75 (*rep17, axe-txe*), 130 (*rep18*), 200 (*rep*_*pLG*1_ *, aac (6*′*)-Ie-aph (2*′′*)-Ia*), 250
a	3567^y#^	144/13/18	315 (*rep17, * *rep*_*pLG*1_ *, pilA*)	75 (*axe-txe, aac (6*′*)-Ie-aph (2*′′*)-Ia*), 240 (rep18)

Letter code of each isolate corresponds to the designation used in Figure 2; nd: not determined; two isolates with the prototype Tn*1546* marked with an asterisk; ^x,y^isolates from the same patients “X” and “Y”; ^#^isolates positive in conjugation.
